# Upper-Body Pitch Control Differentiates Sprint Butterfly Performance in Youth Swimmers: An IMU-Based Analysis

**DOI:** 10.3390/s26102939

**Published:** 2026-05-07

**Authors:** Jinxuan Bao, Shuwen Wang, Yaxuan Huang, Xundian Liu, Yi Peng

**Affiliations:** 1 School of Strength and Conditioning Training, Beijing Sport University, Beijing 100084, China; baojinxuan@bsu.edu.cn; 2 College of Education, Beijing Sport University, Beijing 100084, China; derwangnn@163.com; 3 Sports Coaching College, Beijing Sport University, Beijing 100084, China; yaxuan.huang@bsu.edu.cn (Y.H.); 2023010561@bsu.edu.cn (X.L.)

**Keywords:** biomechanics, swimming, butterfly stroke, kinematics, inertial measurement units, adolescent, athletic performance

## Abstract

Efficient segmental pitch control is critical for butterfly swimming propulsion and alignment, yet its role in youth performance remains unclear. This study quantified head, shoulder, and hip pitch kinematics using wearable inertial measurement units (IMUs) in 41 competitive swimmers (9–11 years). Participants performed two maximal 25-m butterfly trials and were classified into faster and slower groups. Pitch angle, velocity, frequency, time, and pitch deviation index were extracted. Between-group differences were assessed using independent *t*-tests, and associations with performance were examined using Pearson correlations. Faster swimmers exhibited smaller head pitch angles during the Breath phase (*p* < 0.001, *d* = −2.01), along with greater shoulder pitch velocities and frequencies (all *p* < 0.05, *d* = 0.67–1.07). They also demonstrated shorter pitch times and lower pitch deviation indices (all *p* < 0.05, *d* = 0.66–1.92), indicating more efficient and stable movement patterns. In contrast, hip kinematics showed fewer and less consistent differences between groups. Several head and shoulder variables during the Breath phase were moderately correlated with sprint time (*r* = 0.32–0.43, *p* < 0.05). These findings suggest that sprint butterfly performance in youth swimmers is primarily associated with more controlled and stable upper-body pitch motion, particularly during breathing.

## 1. Introduction

Competitive swimming, particularly the butterfly stroke, is one of the most technically demanding disciplines, requiring precise coordination of multiple body segments [1,2]. The efficiency of the butterfly stroke is largely determined by the coordinated pitching motion of the head, shoulder, and hip, which contributes to propulsion, body alignment, and the propagation of the undulatory wave [3,4,5,6]. Effective segmental coordination facilitates force transmission along the body while minimizing unnecessary vertical oscillations and hydrodynamic drag [5,6]. From a dynamical systems perspective, swimming coordination emerges from interactions among task, environmental, and organismic constraints [7,8]. Skilled performance is characterized by stable yet adaptable coordination patterns, often described as attractor states [5,8]. In the present study, these concepts are operationalized using measurable kinematic variables. Temporal parameters (e.g., pitch time and frequency) reflect coordination timing, whereas the pitch deviation index provides an indirect indicator of intra-cycle stability and symmetry. Lower deviation indices and more consistent temporal patterns may therefore indicate greater coordination stability, whereas higher variability may reflect less stable motor organization.

In swimmers aged 9–11 years, coordination patterns are still developing. Neuromuscular control evolves rapidly during late childhood, influenced by growth, training experience, and neurodevelopment [9,10]. This developmental stage is characterized by high neuroplasticity, making it a critical period for establishing efficient movement patterns [11,12]. From a dynamical systems perspective, this adaptability may facilitate the emergence of stable coordination states under appropriate training stimuli [13]. However, immature coordination may also result in less stable and less efficient stroke mechanics, particularly in technically demanding strokes such as the butterfly [9,10].

Despite the recognized importance of segmental coordination, most previous studies have focused on overall stroke mechanics or propulsion characteristics rather than the detailed pitching behavior of individual body segments [14]. The contribution of segment-specific pitch amplitude, temporal characteristics, and movement stability to sprint performance therefore remains unclear, particularly in youth swimmers [15,16]. Understanding these segment-specific pitch characteristics may provide valuable insights for technique optimization and performance development in youth swimmers.

Recent advances in wearable inertial measurement units (IMUs) enable in-water quantification of segmental kinematics, providing an opportunity to address this gap [17,18]. Compared with traditional video-based methods, IMUs provide detailed information on segment orientation and angular motion, allowing continuous and ecologically valid assessment in aquatic environments [19]. However, their application to butterfly biomechanics in youth swimmers remains limited, particularly with respect to segmental pitch coordination [19,20].

Therefore, it remains unclear how segment-specific pitch characteristics—particularly amplitude, temporal parameters, and movement stability—relate to sprint performance in youth butterfly swimmers, and whether these effects differ between breathing phases (Breath vs. No-Breath). To address this gap, the present study quantified segmental pitch kinematics of the head, shoulder, and hip during 25-m butterfly sprint swimming in youth swimmers using IMUs. Differences between performance levels and breathing conditions were examined, and associations with sprint performance were explored. It was hypothesized that faster swimmers would exhibit smaller pitch amplitudes, shorter pitch times, higher frequencies, and lower pitch deviation indices in the head and shoulder segments compared with slower swimmers. These differences were expected to be more pronounced during the Breath phase.

## 2. Materials and Methods

### 2.1. Participants

This study included 41 competitive youth swimmers (age: 9–11 years) from the Wenzhou City Swimming Team, all of whom specialized in the butterfly stroke as their primary or secondary event. Inclusion criteria were: (i) age between 9 and 11 years; and (ii) ≥3 years of structured swimming training experience. According to the participant classification framework proposed by McKay et al. [21], all swimmers were categorized as Tier 2 (Trained/Developmental).

An a priori power analysis (G*Power 3.1, Heinrich-Heine-Universität Düsseldorf, Düsseldorf, Germany; α = 0.05, power = 0.80, effect size *d* = 0.5) indicated a minimum sample size of 34. The final sample (*n* = 41) exceeded this requirement. Written informed consent was obtained from parents or legal guardians, and assent was obtained from all participants. Swimmers with a history of shoulder or lumbar injury within the previous three months were excluded.

The study was approved by the Ethics Committee of Beijing Sport University (Approval No.: 2026088H) and conducted in accordance with the Declaration of Helsinki. Participant characteristics are presented in Table 1.

### 2.2. Performance Classification

Swimming performance was quantified using World Aquatics (formerly FINA) points, which provide a standardized metric for comparing performance across swimmers [22]. Individual FINA points were calculated based on each swimmer’s self-reported personal best time in the 50-m butterfly event. Personal best times were obtained from athletes and coaches and cross-checked against recent competition records, when available, to ensure relevance to the current performance level. This approach provided a more ecologically valid representation of competitive performance compared with short-distance test results.

Participants were divided into a Fast Group (FG, *n* = 23) and a Slow Group (SG, *n* = 18) using a threshold of 300 FINA points. This threshold has been previously used to distinguish developmental-level swimmers and provides a practical criterion for separating performance levels within youth cohorts [22]. In addition, previous research indicates that reaching approximately 300 FINA points in butterfly is typically associated with several years of structured training (≈3.6 years for males and ≈3.3 years for females), suggesting that this threshold may reflect a meaningful stage in skill development rather than an arbitrary cutoff [23]. Therefore, this classification approach was considered appropriate for capturing performance-related differences within this developmental population.

However, it should be acknowledged that dichotomizing a continuous performance variable may lead to some loss of information and does not fully reflect the continuum of individual performance differences. This limitation was taken into account when interpreting the results. Given the developmental stage of the participants, 50 m personal best performance was considered a more stable indicator of competitive level than a single 25 m trial, which may be more influenced by pacing variability and start effects.

### 2.3. Experimental Design

A cross-sectional design was used to examine segmental pitch kinematics during maximal butterfly swimming. Each participant performed two maximal 25-m butterfly sprint trials, separated by a 10-min passive recovery period to minimize fatigue effects [24]. Kinematic data from the two trials were averaged for analysis.

Testing was conducted in January 2026 in a 25-m indoor pool (water temperature: 26.5–27.5 °C). Sessions were scheduled between 09:00 and 11:30 and 14:00 and 17:00 to avoid interference with regular training. Participants were instructed to refrain from eating for at least 2 h before testing while maintaining normal hydration.

### 2.4. Instrumentation and Data Acquisition

Nine-axis IMUs (WitMotion WT901SDCL-BT50, Shenzhen Wit-Motion Technology Co., Ltd., Shenzhen, China; weight: 18 g per unit; sampling frequency: 100 Hz; acceleration range: ±16 g; angular velocity range: ±2000 °·s^−1^; static angle accuracy: 0.05°; onboard 16 GB microSD card storage) were used to capture tri-axial acceleration and angular velocity. Sensors were waterproofed using custom silicone pouches (0.3-mm thermoplastic polyurethane, IP68-rated) and securely attached to three anatomical landmarks using waterproof medical tape (3M Tegaderm Transparent Film Dressing, 1624W; 3M Company, St. Paul, MN, USA): the occipital region (head), the spinous process of T1 (shoulder), and the spinous process of L5 (hip). The total additional mass (sensor + waterproof pouch + tape, approximately 25 g per location) represented less than 0.1% of participants’ body mass, minimizing potential interference with natural swimming kinematics [25,26].

**Figure 1:**
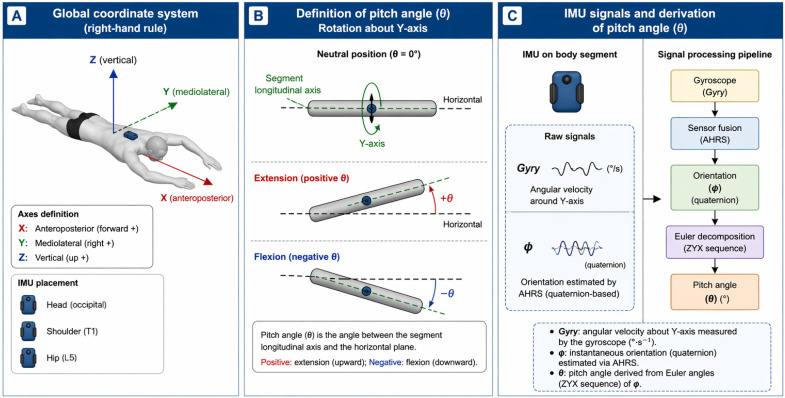
Coordinate system, pitch angle (θ), and IMU signal processing. (**A**) Global coordinate system (X, Y, Z). (**B**) Pitch angle (θ): rotation about the *Y*-axis relative to the horizontal plane (positive: extension; negative: flexion). (**C**) Gyry: angular velocity about *Y*-axis; φ: orientation from AHRS; θ derived from Euler angles (ZYX).

Static calibration was performed with participants standing upright for 5 s prior to data collection to align the sensor coordinate system with the global reference frame. The global coordinate system follows a standard right-hand convention, with axis orientation defined according to the IMU reference frame. Pitch angle (θ) was defined as the rotation of each segment about the mediolateral (Y) axis relative to the global horizontal reference frame, derived from the IMU coordinate system after static calibration. Positive values indicate extension (upward rotation), whereas negative values indicate flexion (downward rotation). As illustrated in Figure 1, θ represents the angle between the longitudinal axis of the segment and the horizontal plane.

Prior to the formal experiment, test–retest reliability was assessed in a subset of 10 swimmers who performed two 25-m butterfly trials 48 h apart, with sensor placement by the same investigator. Intraclass correlation coefficients [ICC(2,1)] were calculated to assess test–retest reliability for key pitch variables, including pitch angle (peak and valley), pitch velocity, pitch time, and pitch frequency. All variables demonstrated good to excellent reliability, with ICC values exceeding 0.80 and corresponding 95% confidence intervals ranging from 0.81 to 0.95. Standard errors of measurement were within acceptable limits, indicating good stability and repeatability of the IMU-based measurements. In addition to reliability, the validity of IMU-derived kinematic measurements in aquatic environments has been supported by previous studies. Specifically, IMUs have demonstrated acceptable agreement with video-based and optoelectronic motion capture systems for estimating segmental orientation and angular kinematics during swimming movements, despite the challenges posed by water turbulence and soft-tissue artefacts [26,27]. Although absolute positional accuracy may be limited compared with gold-standard motion capture systems, IMUs provide sufficiently valid estimates of segmental rotation and temporal characteristics, particularly for within-subject and between-group comparisons in ecological settings. Nevertheless, it should be acknowledged that dynamic aquatic conditions, including fluid resistance and sensor attachment variability, may introduce measurement noise, which should be considered when interpreting the results. These results support the stability and repeatability of the IMU-based measurements for subsequent kinematic analyses.

### 2.5. Experimental Procedure

**Figure 2:**
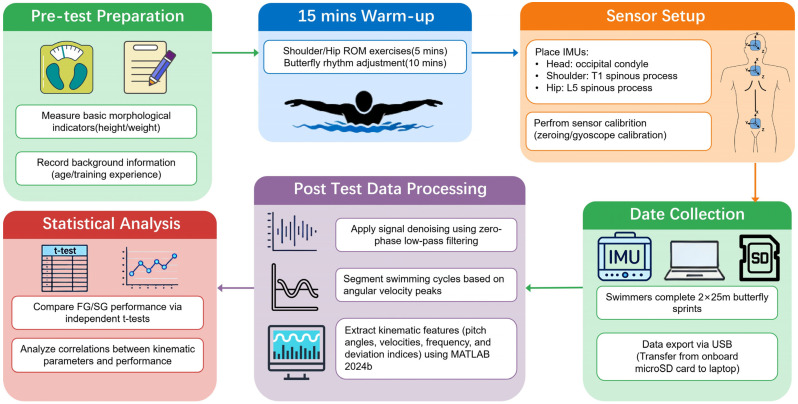
Experimental protocol and data processing workflow.

Upon arrival, participants’ anthropometric characteristics (height and body mass) and training background were recorded. After a 5-min seated rest, resting heart rate was monitored to ensure readiness for testing [28,29]. Participants then completed a standardized 15-min warm-up, including joint mobility exercises and in-water butterfly stroke preparation. Participants were familiar with maximal-effort butterfly swimming due to their regular training, reducing the likelihood of learning effects during testing. IMUs were subsequently attached, followed by static calibration [6]. Each swimmer performed two maximal 25-m butterfly trials, separated by a 10-min passive recovery period. IMU data were recorded continuously during each trial and stored locally on the onboard 16 GB microSD card in offline mode. Upon completion of each trial, data files were transferred from the sensor to a laptop via a USB connection for subsequent offline processing. The mean values from the two trials were used for subsequent analysis. Figure 2 illustrates the experimental design and flow of participants through the study.

### 2.6. Data Processing and Variable Extraction

#### 2.6.1. Data Preprocessing

Raw data were exported via WitMotion software and subsequently processed in MATLAB 2024b (The MathWorks, Inc., Natick, MA, USA). Segment orientation, angular velocity, and acceleration were obtained directly from the sensor’s onboard attitude and heading reference system (AHRS), which applies a quaternion-based Kalman filtering algorithm to fuse tri-axial accelerometer and gyroscope signals. The internal Kalman filtering parameters (e.g., process and measurement noise covariance) are predefined by the manufacturer and not user-adjustable; therefore, sensor fusion was implemented using the device’s default configuration.

To enhance reproducibility, all sensors were initialized under identical conditions, and data were acquired at a sampling frequency of 100 Hz with magnetometer input disabled to minimize magnetic disturbances in the aquatic environment. Orientation estimates were thus computed internally by the sensor prior to data export. The exported Euler angles were imported into MATLAB, and pitch angle was extracted using a ZYX rotation sequence. Given that the analysis focused on sagittal-plane motion, the absence of magnetometer input is unlikely to substantially affect pitch estimation.

According to manufacturer specifications, static orientation accuracy is approximately 0.2°. Potential sources of measurement error include soft-tissue artefacts and sensor attachment variability [26,27,30].

#### 2.6.2. Kinematic Variables

For each segment (head, shoulder, and hip), several pitch-related kinematic variables were extracted:

Pitch Angle: Pitch angle represents the angular displacement of each segment around the mediolateral axis (°), including peak and valley values [31,32].

Pitch Velocity: Pitch velocity represents the rate of angular change (°·s^−1^).

Pitch Frequency: Pitch frequency represents the number of pitch cycles per second (Hz).

Pitch Time: Pitch time represents the duration of a complete pitch cycle (s).

**Figure 3:**
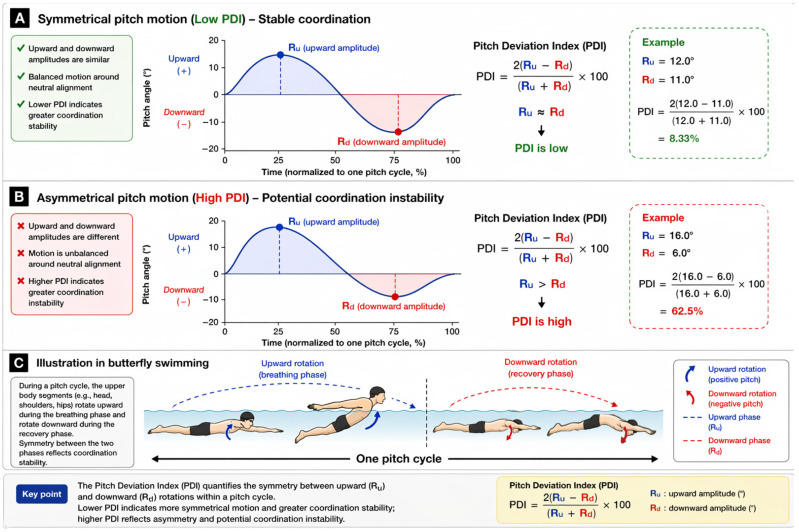
Pitch deviation index (PDI) in butterfly swimming. (**A**) Symmetrical motion (low PDI). (**B**) Asymmetrical motion (high PDI). (**C**) Segmental pitch during breathing and recovery.

Pitch Deviation Index: Pitch deviation index quantifies the asymmetry between upward (R_u_) and downward (R_d_) rotational amplitudes, calculated according to Psycharakis et al. [33,34]. This index has been previously used to quantify asymmetry in cyclic segmental motion and to assess coordination patterns in swimming-related movements. The specific calculation formula is as follows:

$$Pitch\;Deviation\;Index=\frac{2(R_{u}-R_{d})}{\left(R_{u}+R_{d}\right)}\times 100$$

where R_u_ represents the upward rotation amplitude and R_d_ represents the downward rotation amplitude. From a biomechanical perspective, this index reflects the symmetry of segmental oscillation around the neutral alignment and thus provides an indirect indicator of intra-cycle coordination stability. A lower pitch deviation index indicates that upward and downward rotations are more balanced, suggesting a more stable and repeatable movement pattern. In contrast, a higher value reflects greater asymmetry, which may indicate inconsistent motor control, uneven force application, or compensatory movement strategies. A schematic illustration of symmetrical and asymmetrical pitch motion, along with segmental pitch during breathing and recovery, is provided in Figure 3.

This interpretation is particularly relevant in youth swimmers, where neuromuscular coordination is still developing. Increased asymmetry may reflect immature control of segmental motion and reduced ability to stabilize movement against hydrodynamic perturbations. Therefore, the pitch deviation index can be interpreted not only as a kinematic descriptor but also as a proxy for coordination stability and motor control efficiency during cyclic swimming movements.

### 2.7. Statistical Analysis

All statistical analyses were performed using SPSS Statistics (Version 26.0, IBM Corp., Armonk, NY, USA). Data are presented as mean ± standard deviation (SD). Normality and homogeneity of variance were assessed using the Shapiro–Wilk and Levene’s tests, respectively. When the assumption of homogeneity of variance was violated, the Welch correction was applied. Between-group differences in pitch angle, pitch velocity, pitch time, pitch frequency, and pitch deviation index for the head, shoulder, and hip were examined using two-tailed independent-samples *t*-tests (FG vs. SG). Additional comparisons between male and female swimmers were conducted to evaluate potential sex-related confounding effects. Within-subject differences between Breath and No-Breath phases were assessed using paired-samples *t*-tests. Associations between segmental pitch variables and butterfly performance were examined using Pearson correlation coefficients. Correlation strength was interpreted as weak (|*r*| < 0.30), moderate (0.30 ≤ |*r*| < 0.70), or strong (|*r*| ≥ 0.70). All tests were two-tailed, and statistical significance was set at *p* < 0.05. To account for the interdependence of biomechanical variables, a multivariate t-based (MVT) adjustment was applied where appropriate [35,36]. This approach reduces Type I error inflation compared with unadjusted multiple testing while maintaining statistical power. However, given the number of comparisons, residual Type I error cannot be fully excluded, and findings with marginal significance should be interpreted with caution. Effect sizes were calculated for all inferential tests. For *t*-tests, Cohen’s d was used to quantify the magnitude of differences. Effect sizes were interpreted as negligible (<0.20), small (0.20–0.49), moderate (0.50–0.79), large (0.80–1.19), and very large (≥1.20) [37,38]. Statistical results were reported in accordance with APA 7th guidelines, including test statistics with degrees of freedom, exact *p*-values (or *p* < 0.001 where appropriate), and effect sizes [39,40].

## 3. Results

For all between-group comparisons, positive effect sizes indicate greater values in the FG compared to the SG, whereas negative effect sizes indicate smaller values in the FG. For within-subject phase comparisons, positive effect sizes indicate greater values during the Breath phase compared to the No-Breath phase.

### 3.1. Head Pitch Kinematics

**Figure 4:**
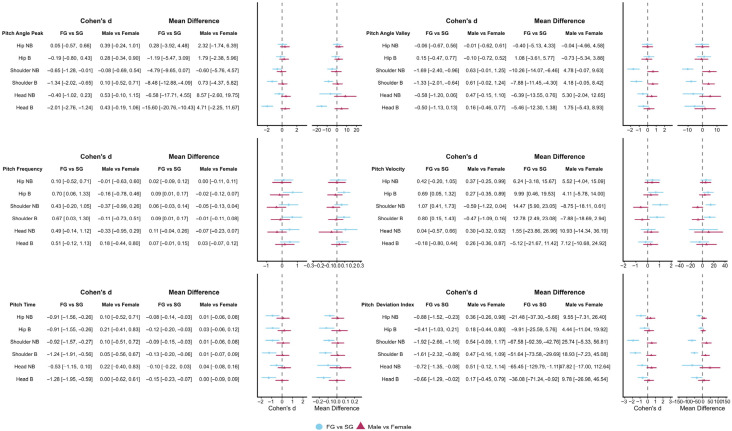
Forest plots summarizing effect sizes (Cohen’s d) for between-group comparisons of pitch kinematic variables. Comparisons include Fast Group versus Slow Group and males versus females across Breath and No-Breath phases. Rows are organized by body segment (head, shoulder, hip) and phase. Abbreviations: Breath (B); No-Breath (NB); Fast Group (FG); Slow Group (SG).

**Figure 5:**
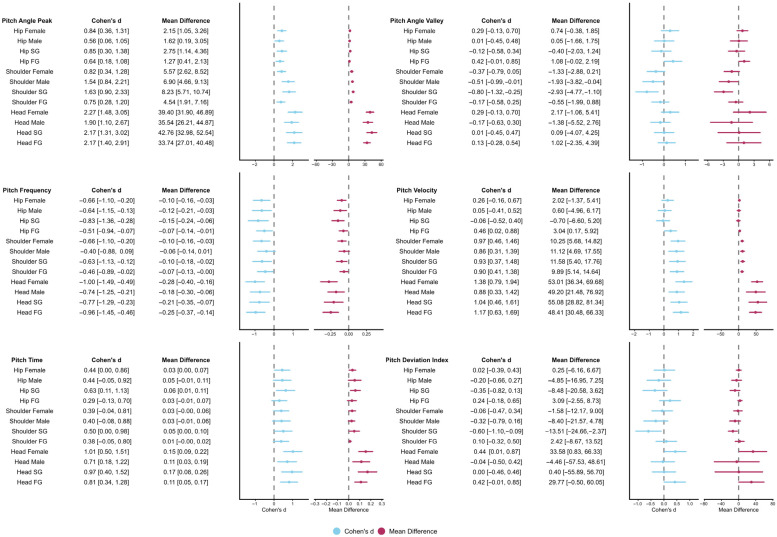
Forest plots summarizing effect sizes (Cohen’s d) for within-subject phase comparisons of pitch kinematic variables. Comparisons reflect differences between Breath and No-Breath phases for performance groups and sexes. Rows are organized by body segment (head, shoulder, hip) and subgroup. Abbreviations: Breath (B); No-Breath (NB); Fast Group (FG); Slow Group (SG).

**Figure 6:**
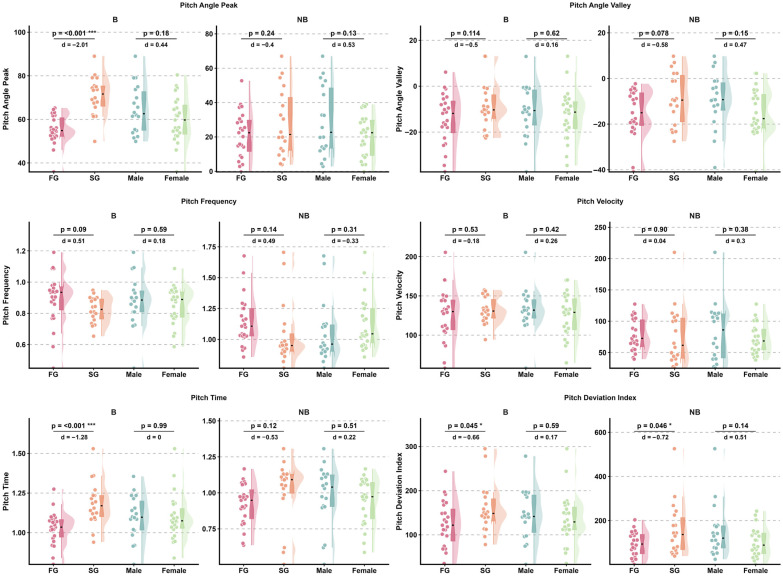
Raincloud plots illustrating between-group differences in head pitch kinematic variables across Fast Group and Slow Group (FG vs. SG) and between sexes (male vs. female) during Breath and No-Breath phases. Abbreviations: Breath (B); No-Breath (NB); Fast Group (FG); Slow Group (SG). Significance levels: *** *p* < 0.001, * *p* < 0.05.

**Figure 7:**
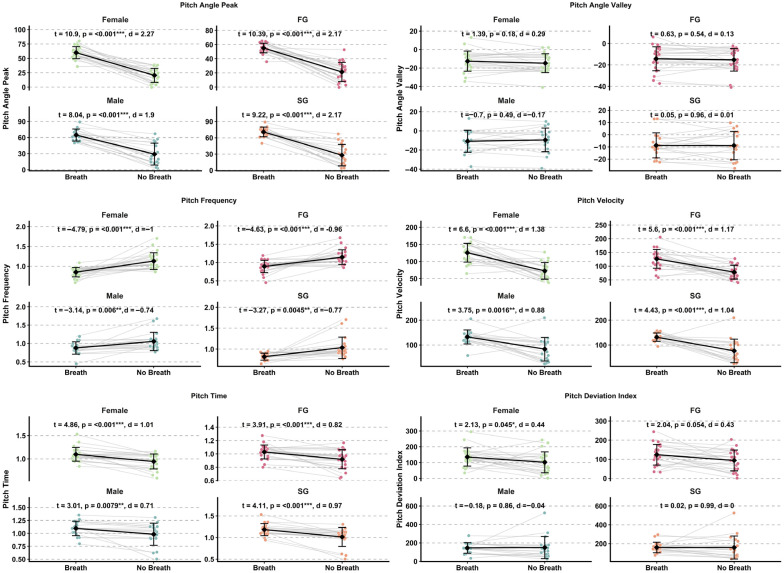
Paired comparison plots showing within-subject differences in head pitch kinematic variables between Breath and No-Breath phases for performance groups (FG, SG) and sexes (male, female). Abbreviations: Breath (B); No-Breath (NB); Fast Group (FG); Slow Group (SG). Significance levels: *** *p* < 0.001, ** *p* < 0.01, * *p* < 0.05.

Effect sizes for head pitch variables are presented in Figure 4 and Figure 5, while detailed kinematic patterns across performance groups, sexes, and phases are illustrated in Figure 6 and Figure 7. Detailed statistical results for all head pitch variables are provided in Supplementary Tables S1, S2, S7 and S8.

Faster swimmers exhibited consistently more controlled head motion, particularly during the Breath phase. Specifically, they showed smaller pitch amplitudes, shorter cycle durations, and lower pitch deviation indices, indicating reduced vertical oscillation and greater intra-cycle stability compared with slower swimmers. This pattern was largely absent during the No-Breath phase, with the exception of a modest reduction in the deviation index in the faster group.

Across all swimmers, the Breath phase was characterized by greater pitch amplitudes and velocities, accompanied by longer pitch times and lower frequencies, reflecting a systematic reorganization of head motion during breathing. No meaningful sex-related differences were observed.

### 3.2. Shoulder Pitch Kinematics

**Figure 8:**
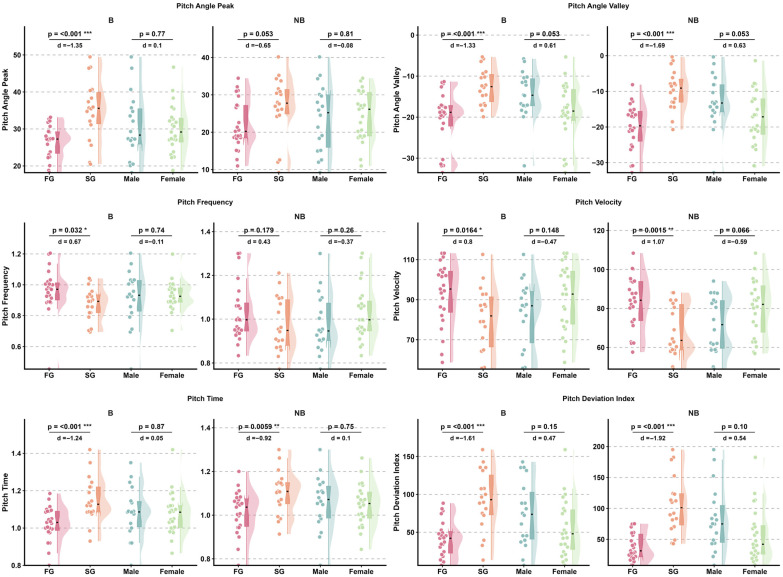
Raincloud plots illustrating between-group differences in shoulder pitch kinematic variables across Fast Group and Slow Group (FG vs. SG) and between sexes (male vs. female) during Breath and No-Breath phases. Abbreviations: Breath (B); No-Breath (NB); Fast Group (FG); Slow Group (SG). Significance levels: *** *p* < 0.001, ** *p* < 0.01, * *p* < 0.05.

**Figure 9:**
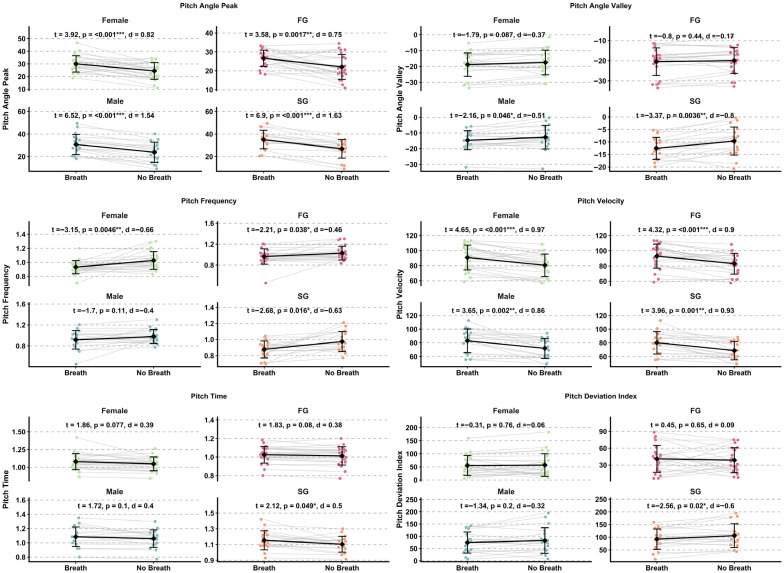
Paired comparison plots showing within-subject differences in shoulder pitch kinematic variables between Breath and No-Breath phases for performance groups (FG, SG) and sexes (male, female). Abbreviations: Breath (B); No-Breath (NB); Fast Group (FG); Slow Group (SG). Significance levels: *** *p* < 0.001, ** *p* < 0.01, * *p* < 0.05.

Effect sizes for shoulder pitch variables are presented in Figure 4 and Figure 5, while detailed kinematic patterns across performance groups, sexes, and phases are illustrated in Figure 8 and Figure 9. Detailed statistical results are provided in Supplementary Tables S3, S4, S7 and S8.

Performance-related differences were most pronounced at the shoulder. Faster swimmers demonstrated a clear pattern of reduced pitch amplitudes, higher angular velocities, higher frequencies (during Breath), shorter pitch times, and consistently lower deviation indices across both phases, indicating a more efficient and temporally organized movement pattern.

In contrast to the head, these differences were robust across both Breath and No-Breath phases, highlighting the shoulder as a key segment for performance differentiation.

Phase comparisons revealed that the Breath phase involved larger and faster shoulder motions but reduced frequency, suggesting a trade-off between movement amplitude and temporal efficiency during breathing. These patterns were consistent across performance groups, with minimal influence of sex.

Importantly, these between-group differences remained consistent when considering sex as a potential confounding factor, as no substantial sex-related differences were observed in the corresponding variables.

### 3.3. Hip Pitch Kinematics

**Figure 10:**
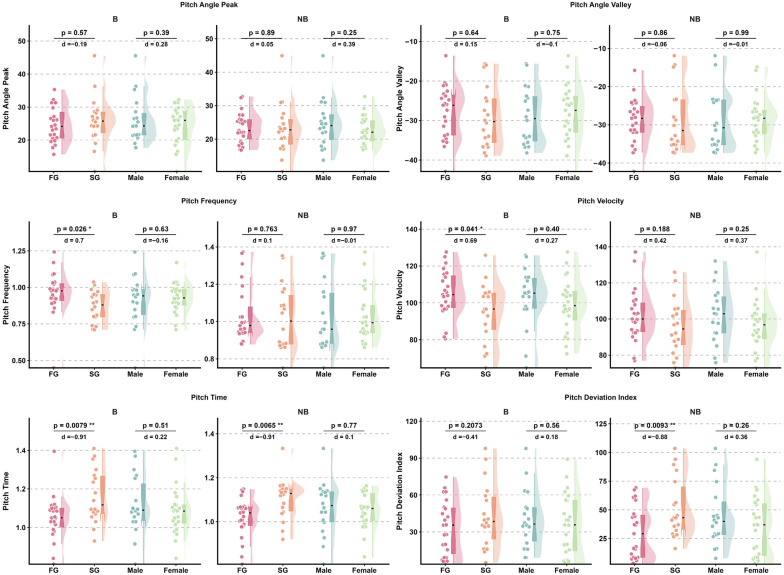
Raincloud plots illustrating between-group differences in hip pitch kinematic variables across Fast Group and Slow Group (FG vs. SG) and between sexes (male vs. female) during Breath and No-Breath phases. Abbreviations: Breath (B); No-Breath (NB); Fast Group (FG); Slow Group (SG). Significance levels: ** *p* < 0.01, * *p* < 0.05.

**Figure 11:**
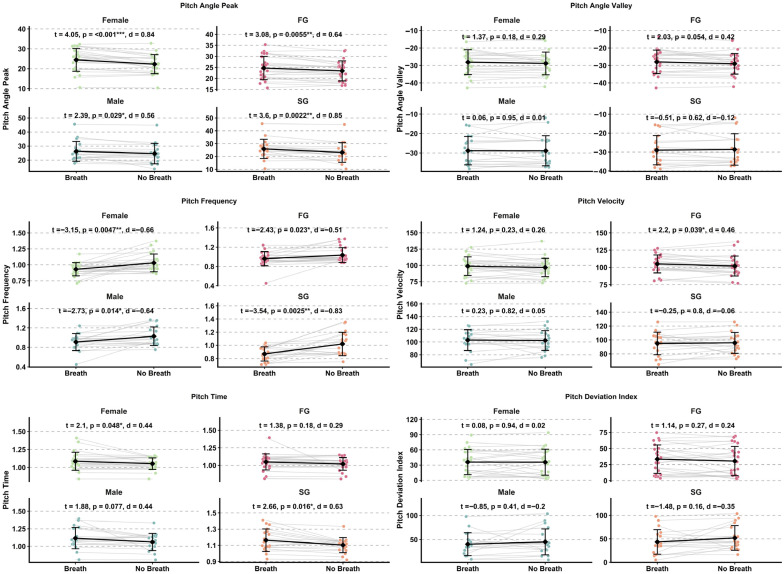
Paired comparison plots showing within-subject differences in hip pitch kinematic variables between Breath and No-Breath phases for performance groups (FG, SG) and sexes (male, female). Abbreviations: Breath (B); No-Breath (NB); Fast Group (FG); Slow Group (SG). Significance levels: *** *p* < 0.001, ** *p* < 0.01, * *p* < 0.05.

Effect sizes for hip pitch variables are presented in Figure 4 and Figure 5, while detailed kinematic patterns across performance groups, sexes, and phases are illustrated in Figure 10 and Figure 11. Detailed statistical results are provided in Supplementary Tables S5–S8.

In contrast to the upper body, hip kinematics showed less pronounced and less consistent differences between performance groups. Faster swimmers demonstrated slightly shorter pitch times and, in the Breath phase, somewhat higher velocities and frequencies, but these effects were smaller and less systematic than those observed in the head and shoulder.

Phase-related changes were still evident, with the Breath phase characterized by greater amplitudes and lower frequencies, mirroring patterns observed in upper-body segments. However, overall, the hip appeared to play a less discriminative role in performance, with weaker and less consistent group differences.

It should also be noted that the absence of statistically significant differences in hip kinematics may, in part, reflect limited statistical power to detect small-to-moderate effects, particularly given the sample size and variability of these measures. Therefore, these findings should be interpreted with caution and not be taken as definitive evidence of no effect.

### 3.4. Kinematic Correlation with 25-m Butterfly Performance

**Figure 12:**
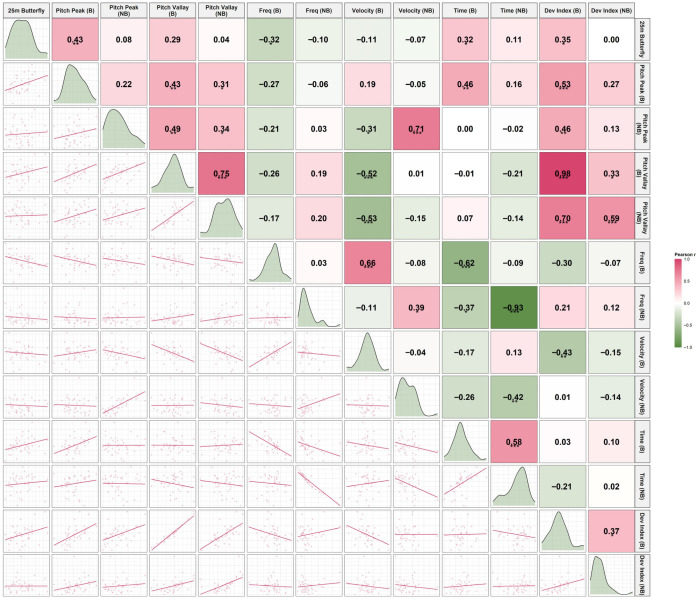
Scatter plot matrix illustrating the associations between head pitch kinematic variables and 25-m butterfly sprint performance. Abbreviations: Breath (B); No-Breath (NB). Significance levels: *** p < 0.001, ** *p* < 0.01, * *p* < 0.05.

**Figure 13:**
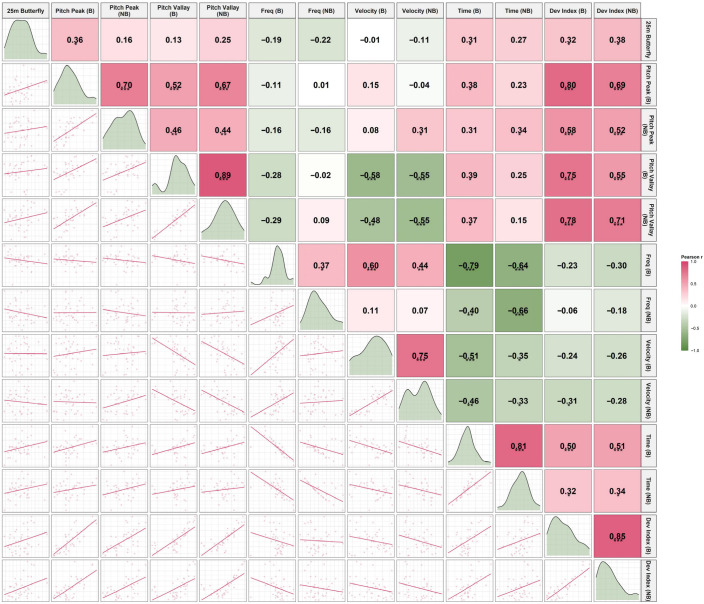
Scatter plot matrix illustrating the associations between shoulder pitch kinematic variables and 25-m butterfly sprint performance. Abbreviations: Breath (B); No-Breath (NB). Significance levels: *** *p* < 0.001, ** *p* < 0.01, * *p* < 0.05.

**Figure 14:**
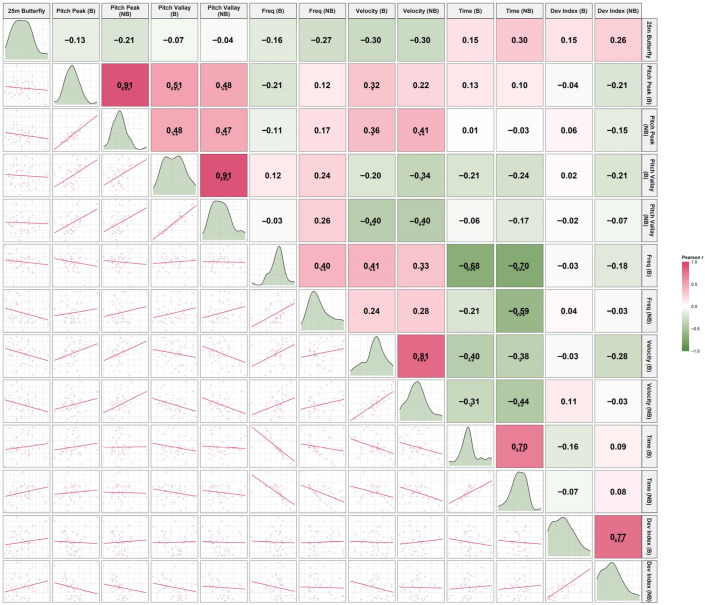
Scatter plot matrix illustrating the associations between hip pitch kinematic variables and 25-m butterfly sprint performance. Abbreviations: Breath (B); No-Breath (NB). Significance levels: *** *p* < 0.001, ** *p* < 0.01, * *p* < 0.05.

The correlation analysis revealed phase-specific associations between segmental pitch characteristics and 25-m butterfly sprint performance, with detailed statistics provided in Table 2 and visual patterns illustrated in Figure 12, Figure 13 and Figure 14.

Correlation analysis revealed clear phase-specific relationships between segmental kinematics and sprint performance.

During the Breath phase, greater head and shoulder pitch amplitudes, longer pitch times, and higher deviation indices were moderately associated with slower sprint performance, whereas higher pitch frequency was associated with faster times. These relationships were largely absent during the No-Breath phase.

Among all variables, the shoulder deviation index (particularly during No-Breath) and head kinematics during Breath showed the most consistent associations with performance. In contrast, hip variables demonstrated weak and non-significant relationships, reinforcing their limited role in performance differentiation.

## 4. Discussion

The present study investigated multi-segment pitch kinematics during butterfly sprint swimming in youth swimmers using wearable (IMUs), providing additional IMU-based evidence on segmental pitch control. These findings support the hypothesis that faster swimmers exhibit more efficient and stable upper-body pitch control, particularly during the Breath phase. Overall, faster swimmers were characterized by more controlled pitch motion and greater movement stability, rather than larger movement amplitudes. This separation between performance classification (50-m PB) and experimental testing (25-m sprint) allowed for controlled kinematic assessment while maintaining ecological validity. Although performance classification was based on 50-m personal best times, the 25-m sprint test was selected to minimize fatigue and isolate kinematic characteristics.

The differences observed in upper-body kinematics may be explained by the combined influence of hydrodynamic alignment and inter-segmental coordination. Faster swimmers exhibited reduced head and shoulder pitch amplitudes during the Breath phase. This likely contributed to improved body alignment and reduced frontal drag [41,42,43]. However, these hydrodynamic mechanisms were not directly measured in the present study and are therefore inferred based on established biomechanical principles and the prior literature. Excessive vertical displacement of the head and upper trunk may disrupt body alignment and increase resistive forces, thereby impairing forward propulsion. Accordingly, the present findings suggest, rather than confirm, that minimizing unnecessary vertical motion during breathing may help maintain propulsion efficiency in youth swimmers [3,44]. The Breath phase emerged as the most performance-relevant component of the stroke cycle. Several head and shoulder variables during this phase showed moderate correlations with sprint performance, whereas variables during the No-Breath phase showed weaker associations. Shoulder variables demonstrated more consistent relationships with performance than head variables, highlighting their potential role in performance differentiation. This pattern likely reflects the inherent challenge of maintaining propulsive continuity while accommodating the postural disruption introduced by breathing [3,29,45].

In addition to amplitude control, faster swimmers exhibited lower pitch deviation indices, particularly at the shoulder, indicating greater intra-cycle consistency and symmetry in segmental motion. From a dynamical systems perspective, reduced asymmetry may reflect more stable coordination patterns. However, coordination stability was not directly quantified and should be interpreted with caution [33,46]. Specifically, the pitch deviation index provides an indirect measure of how consistently upward and downward segmental rotations are organized within each stroke cycle. Lower values may therefore indicate a more balanced and repeatable coordination structure, whereas higher values may reflect greater variability in motor output. Within this framework, more consistent and symmetrical movement patterns may be considered functionally analogous to more stable coordination states, rather than direct evidence of specific dynamical constructs such as attractor states. From a biomechanical perspective, efficient butterfly swimming depends on the smooth propagation of the undulatory wave along body segments. More symmetrical motion may facilitate continuous energy transfer and reduce unnecessary vertical oscillation. In contrast, greater asymmetry—such as excessive upward rotation during breathing—may disrupt this coordination and increase hydrodynamic resistance.

In youth swimmers, such asymmetry may be related to developing neuromuscular control, particularly in regulating transitions between upward and downward movements. Therefore, a lower pitch deviation index may reflect not only mechanical symmetry but also a more consistent temporal organization of inter-segmental coordination. Consequently, improved performance may be associated not only with reduced movement magnitude but also with more stable coordination patterns across the stroke cycle.

Temporal characteristics also differentiated performance levels. Faster swimmers exhibited shorter pitch times at the head and shoulder, indicating more rapid movement cycles. These shorter durations were not consistently accompanied by higher angular velocities, suggesting that temporal efficiency may reflect improved coordination timing rather than simply faster movement. However, it should be noted that coordination was not directly measured, but rather inferred indirectly from kinematic timing variables in the present study. Accordingly, interpretations related to coordination should be considered indicative rather than definitive. This interpretation aligns with previous research indicating that effective swimming technique depends on optimizing the timing and integration of movements while minimizing non-propulsive actions [2,3,29,47]. Improved temporal organization may facilitate smoother energy transfer and reduce mechanical disruption during stroke execution. Additionally, faster swimmers exhibited smaller differences in head pitch velocity between Breath and No-Breath phases, which may indicate smoother integration of the breathing action within the stroke cycle. Such continuity may help preserve undulatory motion and reduce disruptions to propulsion [48,49].

In contrast, hip pitch kinematics exhibited weaker relationships with sprint performance. Although faster swimmers showed slightly shorter hip pitch times, most hip-related variables demonstrated limited between-group differences and weak correlations with performance. This suggests that the trunk-hip segment may function primarily as a stabilizing base rather than a primary performance determinant. Such stability may support proximal-to-distal energy transfer, enabling upper-body segments to accommodate task-specific adjustments such as breathing [4,46,47,50]. Therefore, while hip pitch variables were not primary discriminators of performance in this cohort, they may still contribute indirectly to overall stroke organization and coordination [51].

The present findings carry important implications for the validation and application of wearable sensors in aquatic sports biomechanics. Although underwater motion capture systems remain the gold standard, the present results suggest that IMU-derived pitch metrics are sensitive to performance-related differences in youth swimmers. The moderate-to-large effect sizes observed (*d* = 0.66–2.01) compare favorably with previous video-based studies [11,12], supporting the practical utility of IMUs for field-based technique analysis. However, it is important to acknowledge that IMUs measure orientation relative to the initial calibration position rather than absolute spatial position. Therefore, pitch angles reported in this study represent segmental rotation relative to the static standing reference frame, not absolute orientation in the pool coordinate system. Critically, the capacity to capture continuous kinematics without poolside camera infrastructure addresses a major barrier to widespread biomechanical feedback in youth development programs. Future research should directly compare IMU and optoelectronic systems during butterfly swimming to establish concurrent validity coefficients; however, the present findings support the immediate utility of IMU systems for longitudinal monitoring and talent identification purposes.

The unequal sex distribution between performance groups (FG: 6 males/17 females; SG: 12 males/6 females) reflects the practical reality of talent distribution in this age category rather than a systematic sampling bias. To examine whether this imbalance influenced our conclusions, we conducted supplementary analyses within sex subgroups. These analyses confirmed that the key findings regarding head and shoulder pitch kinematics maintained consistent effect sizes and significance patterns when analyzed separately within males and within females (see Supplementary Tables S1–S6). Furthermore, direct comparisons between males and females revealed no substantial sex-related differences in most kinematic variables at this developmental stage. This pattern is consistent with previous research indicating that, prior to puberty, swimming performance is more strongly influenced by motor coordination and technical proficiency than by sex-related anthropometric factors [9]. Nevertheless, given the unequal sex distribution between groups, the possibility of residual confounding cannot be fully ruled out. Therefore, although the observed differences are most plausibly attributed to variations in neuromuscular control and technical skill development, these findings should be interpreted with appropriate caution.

From a practical perspective, the present findings suggest that coaches should emphasize minimizing excessive head pitch during breathing and improving the temporal coordination of shoulder motion. Specifically, swimmers may benefit from maintaining a more controlled head lift with a forward-oriented gaze, which may help reduce vertical oscillation and maintain body alignment. In addition, drills that promote synchronized undulatory motion—such as body wave exercises and timing-focused butterfly drills—may support more stable inter-segmental coordination. For youth swimmers in particular, these cues should be introduced progressively, with an emphasis on rhythm and coordination rather than force production, to facilitate the development of efficient and repeatable movement patterns.

Several limitations should be acknowledged. First, the cross-sectional design precludes causal inferences, and performance classification based on 50 m personal best times may not fully reflect sprint-specific mechanics assessed in the 25 m test, although this approach was considered more stable for youth swimmers. Second, the modest sample size, particularly within sex subgroups, limits generalizability and sensitivity to detect subtle sex differences. Although sex-related confounding was mitigated by the absence of substantial kinematic differences between males and females, the unequal sex distribution between performance groups warrants cautious interpretation. Although biological maturation was not assessed, the narrow age range (9–11 years) and consistent training backgrounds likely minimized maturation-related confounding effects; however, individual differences in maturation (e.g., Tanner stage) may still influence movement patterns and should be considered in future research. Third, the use of a 25-m sprint test may not reflect pacing strategies or fatigue effects present in longer events. Moreover, only two trials were performed; although averaged to improve stability, residual learning or anxiety effects in young swimmers cannot be fully excluded. Fourth, while IMUs capture kinematic data, they do not provide direct measurements of kinetic variables (e.g., propulsive forces and drag), limiting mechanistic interpretation. In addition, measurement uncertainty associated with IMU-derived kinematics in dynamic aquatic environments should be acknowledged. Factors such as water turbulence, sensor attachment variability, and soft-tissue artefacts may introduce noise and affect signal accuracy, particularly during rapid or high-amplitude movements. Fifth, the analysis was restricted to sagittal-plane motion, and future studies should incorporate three-dimensional kinematics, including roll and yaw. In addition, although a multivariate adjustment procedure was applied, the relatively large number of statistical comparisons may still increase the risk of Type I error, and therefore some findings should be interpreted with caution, particularly those with marginal statistical significance.

## 5. Conclusions

This study examined segmental pitch kinematics of the head, shoulders, and hips during 25-m butterfly sprint swimming in youth swimmers using wearable IMUs. Consistent with our hypothesis, faster swimmers tended to exhibit smaller head and shoulder pitch amplitudes, shorter pitch times, and lower pitch deviation indices, particularly during the Breath phase. These findings suggest that more controlled upper-body oscillation and more stable segmental coordination may be associated with better sprint butterfly performance in youth swimmers.

In contrast, hip pitch variables showed weaker and less consistent relationships with performance, indicating that upper-body kinematic characteristics may play a more prominent role in differentiating sprint ability within this cohort. Overall, IMU-derived kinematic metrics may provide useful indicators for assessing technique and monitoring coordination development in adolescent butterfly swimmers. However, given the correlational nature of the present study, these findings should be interpreted as associative rather than causal. Future longitudinal or intervention-based studies are needed to confirm these relationships.

## Figures and Tables

**Table 1:** Participant characteristics (Mean ± SD). FG, Fast Group; SG, Slow Group.

Variables	All (*n* = 41)	Male (*n* = 18)	Female (*n* = 23)	FG (*n* = 23, 6M/17F)	SG (*n* = 18, 12M/6F)
Age (years)	9.56 ± 0.71	9.78 ± 0.73	9.39 ± 0.66	9.74 ± 0.90	9.33 ± 0.59
Height (cm)	141.35 ± 5.50	142.22 ± 4.76	140.67 ± 6.04	142.35 ± 5.76	140.08 ± 5.03
Weight (kg)	30.95 ± 3.82	32.31 ± 3.85	29.88 ± 3.52	31.41 ± 3.96	30.35 ± 3.66
Butterfly 50-m Time (s)	34.82 ± 2.16	34.02 ± 2.47	35.44 ± 1.68	33.70 ± 1.93	36.25 ± 1.51
FINA Points	286.85 ± 57.23	254.94 ± 56.43	311.83 ± 44.91	329.96 ± 30.28	231.78 ± 28.35

**Table 2:** Pearson correlation coefficients (r) between segmental pitch kinematic variables and 25-m butterfly sprint time, with 95% confidence intervals in brackets. Abbreviations: Breath (B); No-Breath (NB).

Indicator	Head	Shoulder	Hip
Pitch Angle Peak (B)	0.43 [0.14, 0.65]	0.36 [0.06, 0.6]	−0.13 [−0.42, 0.19]
Pitch Angle Peak (NB)	0.08 [−0.23, 0.38]	0.16 [−0.15, 0.45]	−0.21 [−0.48, 0.11]
Pitch Angle Valley (B)	0.29 [−0.02, 0.55]	0.13 [−0.18, 0.42]	−0.07 [−0.37, 0.25]
Pitch Angle Valley (NB)	0.04 [−0.27, 0.34]	0.25 [−0.07, 0.51]	−0.04 [−0.35, 0.27]
Pitch Deviation Index (B)	0.35 [0.04, 0.59]	0.32 [0.02, 0.57]	0.15 [−0.17, 0.44]
Pitch Deviation Index (NB)	0.0 [−0.31, 0.31]	0.38 [0.08, 0.62]	0.25 [−0.06, 0.52]
Pitch Frequency (B)	−0.32 [−0.57, −0.01]	−0.19 [−0.47, 0.12]	−0.16 [−0.45, 0.15]
Pitch Frequency (NB)	−0.1 [−0.4, 0.21]	−0.22 [−0.49, 0.1]	−0.27 [−0.53, 0.05]
Pitch Time (B)	0.32 [0.02, 0.57]	0.31 [0.01, 0.57]	0.15 [−0.16, 0.44]
Pitch Time (NB)	0.11 [−0.2, 0.41]	0.27 [−0.04, 0.53]	0.3 [−0.01, 0.56]
Pitch Velocity (B)	−0.11 [−0.4, 0.21]	−0.01 [−0.32, 0.3]	−0.3 [−0.56, 0.01]
Pitch Velocity (NB)	−0.07 [−0.37, 0.25]	−0.11 [−0.4, 0.21]	−0.3 [−0.56, 0.01]

## Data Availability

Data supporting the findings of this study can be requested from the corresponding author (Y.P.) upon reasonable justification.

## Supplementary Materials

The following supporting information can be downloaded at: https://www.mdpi.com/article/10.3390/s26102939/s1, Table S1: Independent *t*-test Results for Head: FG vs. SG and Male vs. Female; Table S2: Paired *t*-test Results for Head: Breath vs. No-Breath; Table S3: Independent *t*-test Results for Shoulder: FG vs. SG and Male vs. Female; Table S4: Paired *t*-test Results for Shoulder: Breath vs. No-Breath; Table S5: Independent *t*-test Results for Hip: FG vs. SG and Male vs. Female; Table S6: Paired *t*-test Results for Hip: Breath vs. No-Breath. Table S7: Summary of effect sizes (Cohen’s d) for between-group comparisons of pitch kinematic variables by performance level (Fast vs. Slow Group) and sex (Male vs. Female) across Breath and No-Breath phases. Data derived from independent-samples *t*-tests; Table S8: Summary of effect sizes (Cohen’s d) for within-subject phase comparisons (Breath vs. No-Breath) of pitch kinematic variables by performance group (Fast Group, Slow Group) and sex (Male, Female). Data derived from paired-samples *t*-tests.

## Abbreviations

The following abbreviations are used in this manuscript:

IMUs	Inertial measurement units
FG	Fast Group
SG	Slow Group
