# Supplementary material for: Upper-Body Pitch Control Differentiates Sprint Butterfly Performance in Youth Swimmers: An IMU-Based Analysis
Source: Sensors (Basel). 2026 May 7;26(10):2939. doi: 10.3390/s26102939 (PMC13210417; doi:10.3390/s26102939)
Supplement: Supplementary file 1 [file sensors-26-02939-s001.zip › sensors-4245449-supplementary.pdf]

Supplementary Material

1. Supplementary Figures and Tables.

1.1 Supplementary Tables

Supplementary Table S1 Independent t-Test Results for Head: FG vs SG and Male vs Female

Indicator	Condition	FG	SG	t	p	d	Male	Female	t	p	d
Pitch Angle Peak	Breath	55.182±6.657	70.777±8.978	-6.162	<0.001***	-2.011	64.673±11.144	59.959±10.587	1.374	0.178	0.435
Pitch Angle Peak	No Breath	21.437±13.526	28.014±19.743	-1.209	0.237	-0.398	29.133±20.391	20.562±12.206	1.576	0.127	0.526
Pitch Angle Valley	Breath	-14.213±11.234	-8.751±10.333	-1.617	0.114	-0.503	-10.834±11.497	-12.583±10.902	0.495	0.624	0.157
Pitch Angle Valley	No Breath	-15.234±10.574	-8.842±11.664	-1.814	0.0783	-0.578	-9.452±12.334	-14.757±10.258	1.47	0.151	0.473
Pitch Frequency	Breath	0.893±0.168	0.823±0.083	1.747	0.0897	0.51	0.877±0.166	0.851±0.119	0.548	0.588	0.179
Pitch Frequency	No Breath	1.146±0.199	1.035±0.254	1.527	0.137	0.495	1.055±0.249	1.130±0.212	-1.027	0.312	-0.33
Pitch Velocity	Breath	126.464±34.037	131.587±16.896	-0.629	0.533	-0.184	132.707±28.295	125.587±27.372	0.811	0.423	0.256
Pitch Velocity	No Breath	78.058±25.369	76.506±47.231	0.126	0.901	0.042	83.507±47.310	72.580±24.168	0.893	0.381	0.302
Pitch Time	Breath	1.029±0.104	1.184±0.139	-3.925	<0.001***	-1.28	1.097±0.140	1.097±0.147	-0.01	0.992	-0.003
Pitch Time	No Breath	0.919±0.143	1.014±0.220	-1.59	0.123	-0.526	0.983±0.214	0.943±0.160	0.661	0.513	0.216
Pitch Deviation Index	Breath	123.907±53.865	159.986±56.030	-2.081	0.045*	-0.658	145.231±57.738	135.454±57.488	0.539	0.593	0.17
Pitch Deviation Index	No Breath	94.133±54.610	159.585±122.543	-2.108	0.047*	-0.722	149.693±120.017	101.875±66.124	1.519	0.141	0.511

Note：Values are presented as mean±SD. FG: Faster Group; SG: Slower Group; P: P value($P < 0.05$, ** $P < 0.01$, *** $P < 0.001$); B: Breathing; NB: Non-Breathing; FG: Fast Group; SG: Slow Group;*

Supplementary Table S2 Paired t-Test Results for Head: Breath vs No Breath

Indicator	Group	Breath	No Breath	t	p	d
Pitch Angle Peak	FG	55.182±6.657	21.437±13.526	10.387	<0.001***	2.166
Pitch Angle Peak	SG	70.777±8.978	28.014±19.743	9.224	<0.001***	2.174
Pitch Angle Peak	Male	64.673±11.144	29.133±20.391	8.041	<0.001***	1.895
Pitch Angle Peak	Female	59.959±10.587	20.562±12.206	10.901	<0.001***	2.273
Pitch Angle Valley	FG	-14.213±11.234	-15.234±10.574	0.628	0.536	0.131
Pitch Angle Valley	SG	-8.751±10.333	-8.842±11.664	0.046	0.963	0.011
Pitch Angle Valley	Male	-10.834±11.497	-9.452±12.334	-0.704	0.491	-0.166
Pitch Angle Valley	Female	-12.583±10.902	-14.757±10.258	1.395	0.177	0.291
Pitch Frequency	FG	0.893±0.168	1.146±0.199	-4.625	<0.001***	-0.964
Pitch Frequency	SG	0.823±0.083	1.035±0.254	-3.272	0.004**	-0.771
Pitch Frequency	Male	0.877±0.166	1.055±0.249	-3.138	0.006**	-0.74
Pitch Frequency	Female	0.851±0.119	1.13±0.212	-4.785	<0.001***	-0.998
Pitch Velocity	FG	126.464±34.037	78.058±25.369	5.601	<0.001***	1.168
Pitch Velocity	SG	131.587±16.896	76.506±47.231	4.426	<0.001***	1.043
Pitch Velocity	Male	132.707±28.295	83.507±47.31	3.745	0.002**	0.883
Pitch Velocity	Female	125.587±27.372	72.58±24.168	6.595	<0.001***	1.375
Pitch Time	FG	1.029±0.104	0.919±0.143	3.911	<0.001***	0.815
Pitch Time	SG	1.184±0.139	1.014±0.22	4.108	<0.001***	0.968
Pitch Time	Male	1.097±0.14	0.983±0.214	3.011	0.008**	0.71
Pitch Time	Female	1.097±0.147	0.943±0.16	4.865	<0.001***	1.014
Pitch Deviation Index	FG	123.907±53.865	94.133±54.61	2.04	0.054	0.425
Pitch Deviation Index	SG	159.986±56.03	159.585±122.543	0.015	0.988	0.004
Pitch Deviation Index	Male	145.231±57.738	149.693±120.017	-0.177	0.861	-0.042
Pitch Deviation Index	Female	135.454±57.488	101.875±66.124	2.126	0.045*	0.443

Note：Values are presented as mean ±SD. FG: Faster Group; SG: Slower Group; P value(P < 0.05, ** P < 0.01, *** P < 0.001); B: Breathing; NB: Non-Breathing; FG: Fast Group; SG: Slow Group;*

Supplementary Table S3 Independent t-Test Results for Shoulder: FG vs SG and Male vs Female

Indicator	Condition	FG	SG	t	p	d	Male	Female	t	p	d
Pitch Angle Peak	Breath	26.617±4.296	35.099±8.203	-3.98	<0.001***	-1.345	30.748±8.877	30.021±6.507	0.291	0.773	0.095
Pitch Angle Peak	No Breath	22.082±6.583	26.872±8.284	-2.007	0.053	-0.65	23.849±8.971	24.448±6.674	-0.237	0.815	-0.077
Pitch Angle Valley	Breath	-20.435±6.860	-12.558±4.383	-4.465	<0.001***	-1.333	-14.629±6.047	-18.814±7.354	1.999	0.053	0.614
Pitch Angle Valley	No Breath	-19.885±6.434	-9.625±5.592	-5.456	<0.001***	-1.687	-12.698±7.515	-17.480±7.730	1.997	0.0532	0.626
Pitch Frequency	Breath	0.964±0.147	0.876±0.105	2.224	0.032*	0.672	0.917±0.178	0.932±0.094	-0.332	0.743	-0.112
Pitch Frequency	No Breath	1.030±0.131	0.975±0.125	1.371	0.179	0.429	0.979±0.134	1.027±0.126	-1.156	0.255	-0.366
Pitch Velocity	Breath	92.979±15.794	80.197±16.392	2.518	0.016*	0.796	82.947±17.245	90.826±16.546	-1.478	0.148	-0.467
Pitch Velocity	No Breath	83.087±13.649	68.614±13.292	3.419	0.002**	1.073	71.825±14.536	80.574±14.851	-1.895	0.066	-0.595
Pitch Time	Breath	1.024±0.090	1.154±0.121	-3.807	<0.001***	-1.241	1.085±0.136	1.078±0.114	0.167	0.868	0.054
Pitch Time	No Breath	1.013±0.098	1.104±0.100	-2.927	0.006**	-0.924	1.059±0.124	1.048±0.097	0.321	0.750	0.104
Pitch Deviation Index	Breath	41.086±23.943	92.722±40.062	-4.834	<0.001***	-1.614	74.372±43.112	55.447±37.904	1.47	0.151	0.47
Pitch Deviation Index	No Breath	38.662±22.145	106.237±46.950	-5.636	<0.001***	-1.921	82.767±52.216	57.029±43.344	1.686	0.101	0.543

Note：Values are presented as mean±SD. FG: Faster Group; SG: Slower Group; P: P value(P < 0.05, ** P < 0.01, *** P < 0.001); B: Breathing; NB: Non-Breathing; FG: Fast Group; SG: Slow Group;*

Supplementary Table S4 Paired t-Test Results for Shoulder: Breath vs No Breath

Indicator	Group	Breath	No Breath	t	p	d
Pitch Angle Peak	FG	26.617±4.296	22.082±6.583	3.58	0.002**	0.746
Pitch Angle Peak	SG	35.099±8.203	26.872±8.284	6.9	<0.001***	1.626
Pitch Angle Peak	Male	30.748±8.877	23.849±8.971	6.516	<0.001***	1.536
Pitch Angle Peak	Female	30.021±6.507	24.448±6.674	3.918	<0.001***	0.817
Pitch Angle Valley	FG	-20.435±6.86	-19.885±6.434	-0.795	0.435	-0.166
Pitch Angle Valley	SG	-12.558±4.383	-9.625±5.592	-3.373	0.004**	-0.795
Pitch Angle Valley	Male	-14.629±6.047	-12.698±7.515	-2.155	0.046*	-0.508
Pitch Angle Valley	Female	-18.814±7.354	-17.48±7.73	-1.788	0.088	-0.373
Pitch Frequency	FG	0.964±0.147	1.03±0.131	-2.207	0.038*	-0.46

Indicator	Group	Breath	No Breath	t	p	d
Pitch Frequency	SG	0.876±0.105	0.975±0.125	-2.679	0.016*	-0.632
Pitch Frequency	Male	0.917±0.178	0.979±0.134	-1.696	0.108	-0.4
Pitch Frequency	Female	0.932±0.094	1.027±0.126	-3.153	0.005**	-0.658
Pitch Velocity	FG	92.979±15.794	83.087±13.649	4.318	<0.001***	0.9
Pitch Velocity	SG	80.197±16.392	68.614±13.292	3.955	0.001**	0.932
Pitch Velocity	Male	82.947±17.245	71.825±14.536	3.648	0.002**	0.86
Pitch Velocity	Female	90.826±16.546	80.574±14.851	4.653	<0.001***	0.97
Pitch Time	FG	1.024±0.09	1.013±0.098	1.833	0.0803	0.382
Pitch Time	SG	1.154±0.121	1.104±0.1	2.115	0.0495*	0.499
Pitch Time	Male	1.085±0.136	1.059±0.124	1.717	0.104	0.405
Pitch Time	Female	1.078±0.114	1.048±0.097	1.857	0.077	0.387
Pitch Deviation Index	FG	41.086±23.943	38.662±22.145	0.453	0.655	0.095
Pitch Deviation Index	SG	92.722±40.062	106.237±46.95	-2.558	0.020*	-0.603
Pitch Deviation Index	Male	74.372±43.112	82.767±52.216	-1.345	0.196	-0.317
Pitch Deviation Index	Female	55.447±37.904	57.029±43.344	-0.31	0.759	-0.065

Note：Values are presented as mean±SD. FG: Faster Group; SG: Slower Group; P: P value(P < 0.05, ** P < 0.01, *** P < 0.001); B: Breathing; NB: Non-Breathing; FG: Fast Group; SG: Slow Group;*

Supplementary Table S5 Independent t-Test Results for Hip: FG vs SG and Male vs Female

Indicator	Condition	FG	SG	t	p	d	Male	Female	t	p	d
Pitch Angle Peak	Breath	24.716±5.307	25.909±7.54	-0.57	0.573	-0.187	26.243±7.04	24.454±5.743	0.874	0.388	0.282
Pitch Angle Peak	No Breath	23.443±4.565	23.16±7.675	0.138	0.891	0.046	24.621±7.266	22.3±4.809	1.169	0.252	0.387
Pitch Angle Valley	Breath	-27.954±6.734	-29.035±7.759	0.469	0.642	0.15	-28.839±7.304	-28.108±7.137	-0.321	0.75	-0.101
Pitch Angle Valley	No Breath	-29.04±5.893	-28.639±8.317	-0.173	0.864	-0.057	-28.886±7.756	-28.847±6.464	-0.017	0.986	-0.006
Pitch Frequency	Breath	0.961±0.148	0.869±0.106	2.315	0.026*	0.7	0.908±0.175	0.931±0.103	-0.483	0.633	-0.162
Pitch Frequency	No Breath	1.036±0.153	1.02±0.177	0.303	0.763	0.097	1.028±0.192	1.03±0.14	-0.044	0.965	-0.014
Pitch Velocity	Breath	105.029±12.989	95.035±16.209	2.134	0.041*	0.69	102.947±16.334	98.837±14.27	0.845	0.404	0.27
Pitch Velocity	No Breath	101.984±14.444	95.74±15.023	1.343	0.188	0.425	102.342±15.609	96.817±14.094	1.173	0.249	0.374
Pitch Time	Breath	1.049±0.115	1.164±0.139	-2.828	0.008**	-0.911	1.116±0.152	1.086±0.126	0.669	0.508	0.215
Pitch Time	No Breath	1.02±0.09	1.103±0.093	-2.887	0.007**	-0.912	1.062±0.121	1.052±0.081	0.29	0.774	0.096
Pitch Deviation Index	Breath	33.499±22.092	43.41±26.215	-1.286	0.207	-0.413	40.342±23.89	35.899±24.781	0.581	0.565	0.182
Pitch Deviation Index	No Breath	30.407±22.792	51.888±26.166	-2.759	0.009**	-0.883	45.193±27.037	35.646±25.566	1.149	0.258	0.364

Note：Values are presented as mean±SD. FG: Faster Group; SG: Slower Group; P: P value($P < 0.05$, ** $P < 0.01$, *** $P < 0.001$); B: Breathing; NB: Non-Breathing; FG: Fast Group; SG: Slow Group;*

Supplementary Table S6 Paired t-Test Results for Hip: Breath vs No Breath

Indicator	Group	Breath	No Breath	t	p	d
Pitch Angle Peak	FG	24.716±5.307	23.443±4.565	3.075	0.006**	0.641
Pitch Angle Peak	SG	25.909±7.540	23.160±7.675	3.6	0.002**	0.848
Pitch Angle Peak	Male	26.243±7.040	24.621±7.266	2.391	0.027*	0.564
Pitch Angle Peak	Female	24.454±5.743	22.300±4.809	4.047	<0.001***	0.844
Pitch Angle Valley	FG	-27.954±6.734	-29.040±5.893	2.032	0.054	0.424
Pitch Angle Valley	SG	-29.035±7.759	-28.639±8.317	-0.51	0.616	-0.12
Pitch Angle Valley	Male	-28.839±7.304	-28.886±7.756	0.058	0.954	0.014
Pitch Angle Valley	Female	-28.108±7.137	-28.847±6.464	1.374	0.183	0.287
Pitch Frequency	FG	0.961±0.148	1.036±0.153	-2.435	0.024*	-0.508
Pitch Frequency	SG	0.869±0.106	1.020±0.177	-3.536	0.003**	-0.834
Pitch Frequency	Male	0.908±0.175	1.028±0.192	-2.734	0.014*	-0.644
Pitch Frequency	Female	0.931±0.103	1.030±0.140	-3.147	0.005**	-0.656
Pitch Velocity	FG	105.029±12.989	101.984±14.444	2.2	0.039*	0.459
Pitch Velocity	SG	95.035±16.209	95.740±15.023	-0.252	0.804	-0.059
Pitch Velocity	Male	102.947±16.334	102.342±15.609	0.229	0.821	0.054
Pitch Velocity	Female	98.837±14.270	96.817±14.094	1.236	0.230	0.258
Pitch Time	FG	1.049±0.115	1.020±0.090	1.382	0.181	0.288
Pitch Time	SG	1.164±0.139	1.103±0.093	2.662	0.016*	0.627
Pitch Time	Male	1.116±0.152	1.062±0.121	1.884	0.077	0.444
Pitch Time	Female	1.086±0.126	1.052±0.081	2.098	0.048*	0.438
Pitch Deviation Index	FG	33.499±22.092	30.407±22.792	1.137	0.268	0.237
Pitch Deviation Index	SG	43.410±26.215	51.888±26.166	-1.478	0.158	-0.348
Pitch Deviation Index	Male	40.342±23.890	45.193±27.037	-0.846	0.409	-0.199
Pitch Deviation Index	Female	35.899±24.781	35.646±25.566	0.082	0.936	0.017

Note：Values are presented as mean±SD. FG: Faster Group; SG: Slower Group; P: P value(P < 0.05, ** P < 0.01, *** P < 0.001); B: Breathing; NB: Non-Breathing; FG: Fast Group; SG: Slow Group;*

Supplementary Table S7 Summary of effect sizes (Cohen's d) for within-subject phase comparisons (Breath vs. No-Breath) of pitch kinematic variables by

performance group (Fast Group, Slow Group) and sex (Male, Female). Data derived from paired-samples t-tests.

Segment	Indicator	Condition	Cohen's d with 95%CI		Mean Difference with 95%CI	
			FG vs.SG	Male vs.Femal	FG vs.SG	Male vs.Femal
Head	Pitch Angle Peak	Breath	-2.01 (-2.76, -1.24)	0.43 (-0.19, 1.06)	-15.60 (-20.76, -10.43)	4.71 (-2.25, 11.67)
		No Breath	-0.40 (-1.02, 0.23)	0.53 (-0.10, 1.15)	-6.58 (-17.71, 4.55)	8.57 (-2.6, 19.75)
	Pitch Angle Valley	Breath	-0.50 (-1.13, 0.13)	0.16 (-0.46, 0.77)	-5.46 (-12.30, 1.38)	1.75 (-5.43, 8.93)
		No Breath	-0.58 (-1.20, 0.06)	0.47 (-0.15, 1.10)	-6.39 (-13.55, 0.76)	5.30 (-2.04, 12.65)
	Pitch Frequency	Breath	0.51 (-0.12, 1.13)	0.18 (-0.44, 0.80)	0.07 (-0.01, 0.15)	0.03 (-0.07, 0.12)
		No Breath	0.49 (-0.14, 1.12)	-0.33 (-0.95, 0.29)	0.11 (-0.04, 0.26)	-0.07 (-0.23, 0.07)
	Pitch Velocity	Breath	-0.18 (-0.80, 0.44)	0.26 (-0.36, 0.87)	-5.12 (-21.67, 11.42)	7.12 (-10.68, 24.92)
		No Breath	0.04 (-0.57, 0.66)	0.30 (-0.32, 0.92)	1.55 (-23.86, 26.96)	10.93 (-14.34, 36.19)
	Pitch Time	Breath	-1.28 (-1.95, -0.59)	-0.00 (-0.62, 0.61)	-0.15 (-0.23, -0.07)	0.00 (-0.09, 0.09)
		No Breath	-0.53 (-1.15, 0.10)	0.22 (-0.40, 0.83)	-0.10 (-0.22, 0.03)	0.04 (-0.08, 0.16)
	Pitch Deviation Index	Breath	-0.66 (-1.29, -0.02)	0.17 (-0.45, 0.79)	-36.08 (-71.24, -0.92)	9.78 (-26.98, 46.54)
		No Breath	-0.72 (-1.35, -0.08)	0.51 (-0.12, 1.14)	-65.45 (-129.79, -1.11)	47.82 (-17.0, 112.64)
Shoulder	Pitch Angle Peak	Breath	-1.34 (-2.02, -0.65)	0.10 (-0.52, 0.71)	-8.48 (-12.88, -4.09)	0.73 (-4.37, 5.82)
		No Breath	-0.65 (-1.28, -0.01)	-0.08 (-0.69, 0.54)	-4.79 (-9.65, 0.07)	-0.60 (-5.76, 4.57)
	Pitch Angle Valley	Breath	-1.33 (-2.01, -0.64)	0.61 (-0.02, 1.24)	-7.88 (-11.45, -4.30)	4.18 (-0.05, 8.42)
		No Breath	-1.69 (-2.40, -0.96)	0.63 (-0.01, 1.25)	-10.26 (-14.07, -6.46)	4.78 (-0.07, 9.63)
	Pitch Frequency	Breath	0.67 (0.03, 1.30)	-0.11 (-0.73, 0.51)	0.09 (0.01, 0.17)	-0.01 (-0.11, 0.08)
		No Breath	0.43 (-0.20, 1.05)	-0.37 (-0.99, 0.26)	0.06 (-0.03, 0.14)	-0.05 (-0.13, 0.04)
	Pitch Velocity	Breath	0.80 (0.15, 1.43)	-0.47 (-1.09, 0.16)	12.78 (2.49, 23.08)	-7.88 (-18.69, 2.94)
		No Breath	1.07 (0.41, 1.73)	-0.59 (-1.22, 0.04)	14.47 (5.90, 23.05)	-8.75 (-18.11, 0.61)
	Pitch Time	Breath	-1.24 (-1.91, -0.56)	0.05 (-0.56, 0.67)	-0.13 (-0.20, -0.06)	0.01 (-0.07, 0.09)
		No Breath	-0.92 (-1.57, -0.27)	0.10 (-0.51, 0.72)	-0.09 (-0.15, -0.03)	0.01 (-0.06, 0.08)
	Pitch Deviation Index	Breath	-1.61 (-2.32, -0.89)	0.47 (-0.16, 1.09)	-51.64 (-73.58, -29.69)	18.93 (-7.23, 45.08)
		No Breath	-1.92 (-2.66, -1.16)	0.54 (-0.09, 1.17)	-67.58 (-92.39, -42.76)	25.74 (-5.33, 56.81)
Hip	Pitch Angle Peak	Breath	-0.19 (-0.80, 0.43)	0.28 (-0.34, 0.90)	-1.19 (-5.47, 3.09)	1.79 (-2.38, 5.96)
		No Breath	0.05 (-0.57, 0.66)	0.39 (-0.24, 1.01)	0.28 (-3.92, 4.48)	2.32 (-1.74, 6.39)
	Pitch Angle Valley	Breath	0.15 (-0.47, 0.77)	-0.1 (-0.72, 0.52)	1.08 (-3.61, 5.77)	-0.73 (-5.34, 3.88)
		No Breath	-0.06 (-0.67, 0.56)	-0.01 (-0.62, 0.61)	-0.4 (-5.13, 4.33)	-0.04 (-4.66, 4.58)
	Pitch Frequency	Breath	0.70 (0.06, 1.33)	-0.16 (-0.78, 0.46)	0.09 (0.01, 0.17)	-0.02 (-0.12, 0.07)

Segment	Indicator	Condition	Cohen's d with 95%CI		Mean Difference with 95%CI	
			FG vs.SG	Male vs.Femal	FG vs.SG	Male vs.Femal
		No Breath	0.10 (-0.52, 0.71)	-0.01 (-0.63, 0.60)	0.02 (-0.09, 0.12)	-0.0 (-0.11, 0.11)
	Pitch Velocity	Breath	0.69 (0.05, 1.32)	0.27 (-0.35, 0.89)	9.99 (0.46, 19.53)	4.11 (-5.78, 14.0)
		No Breath	0.42 (-0.20, 1.05)	0.37 (-0.25, 0.99)	6.24 (-3.18, 15.67)	5.52 (-4.04, 15.09)
	Pitch Time	Breath	-0.91 (-1.55, -0.26)	0.21 (-0.41, 0.83)	-0.12 (-0.2, -0.03)	0.03 (-0.06, 0.12)
		No Breath	-0.91 (-1.56, -0.26)	0.10 (-0.52, 0.71)	-0.08 (-0.14, -0.03)	0.01 (-0.06, 0.08)
	Pitch Deviation Index	Breath	-0.41 (-1.03, 0.21)	0.18 (-0.44, 0.80)	-9.91 (-25.59, 5.76)	4.44 (-11.04, 19.92)
		No Breath	-0.88 (-1.52, -0.23)	0.36 (-0.26, 0.98)	-21.48 (-37.3, -5.66)	9.55 (-7.31, 26.4)

Supplementary Table S8 Summary of effect sizes (Cohen's d) for within-subject phase comparisons (Breath vs. No-Breath) of pitch kinematic variables by performance group (Fast Group, Slow Group) and sex (Male, Female). Data derived from paired-samples t-tests.

Indicator	Segment	Group	Mean Difference with 95%CI	Cohen's d with 95%CI
Pitch Angle Peak	Head	FG	33.74 [27.01, 40.48]	2.17 [1.40, 2.91]
Pitch Angle Peak	Head	Female	39.40 [31.90, 46.89]	2.27 [1.48, 3.05]
Pitch Angle Peak	Head	Male	35.54 [26.21, 44.87]	1.90 [1.10, 2.67]
Pitch Angle Peak	Head	SG	42.76 [32.98, 52.54]	2.17 [1.31, 3.02]
Pitch Angle Peak	Hip	FG	1.27 [0.41, 2.13]	0.64 [0.18, 1.08]
Pitch Angle Peak	Hip	Female	2.15 [1.05, 3.26]	0.84 [0.36, 1.31]
Pitch Angle Peak	Hip	Male	1.62 [0.19, 3.05]	0.56 [0.06, 1.05]
Pitch Angle Peak	Hip	SG	2.75 [1.14, 4.36]	0.85 [0.30, 1.38]
Pitch Angle Peak	Shoulder	FG	4.54 [1.91, 7.16]	0.75 [0.28, 1.20]
Pitch Angle Peak	Shoulder	Female	5.57 [2.62, 8.52]	0.82 [0.34, 1.28]
Pitch Angle Peak	Shoulder	Male	6.90 [4.66, 9.13]	1.54 [0.84, 2.21]
Pitch Angle Peak	Shoulder	SG	8.23 [5.71, 10.74]	1.63 [0.90, 2.33]
Pitch Angle Valley	Head	FG	1.02 [-2.35, 4.39]	0.13 [-0.28, 0.54]
Pitch Angle Valley	Head	Female	2.17 [-1.06, 5.41]	0.29 [-0.13, 0.70]
Pitch Angle Valley	Head	Male	-1.38 [-5.52, 2.76]	-0.17 [-0.63, 0.30]
Pitch Angle Valley	Head	SG	0.09 [-4.07, 4.25]	0.01 [-0.45, 0.47]
Pitch Angle Valley	Hip	FG	1.08 [-0.02, 2.19]	0.42 [-0.01, 0.85]
Pitch Angle Valley	Hip	Female	0.74 [-0.38, 1.85]	0.29 [-0.13, 0.70]
Pitch Angle Valley	Hip	Male	0.05 [-1.66, 1.75]	0.01 [-0.45, 0.48]
Pitch Angle Valley	Hip	SG	-0.40 [-2.03, 1.24]	-0.12 [-0.58, 0.34]
Pitch Angle Valley	Shoulder	FG	-0.55 [-1.99, 0.88]	-0.17 [-0.58, 0.25]
Pitch Angle Valley	Shoulder	Female	-1.33 [-2.88, 0.21]	-0.37 [-0.79, 0.05]
Pitch Angle Valley	Shoulder	Male	-1.93 [-3.82, -0.04]	-0.51 [-0.99, -0.01]
Pitch Angle Valley	Shoulder	SG	-2.93 [-4.77, -1.10]	-0.80 [-1.32, -0.25]
Pitch Deviation Index	Head	FG	29.77 [-0.50, 60.05]	0.42 [-0.01, 0.85]
Pitch Deviation Index	Head	Female	33.58 [0.83, 66.33]	0.44 [0.01, 0.87]
Pitch Deviation Index	Head	Male	-4.46 [-57.53, 48.61]	-0.04 [-0.50, 0.42]
Pitch Deviation Index	Head	SG	0.40 [-55.89, 56.70]	0.00 [-0.46, 0.46]
Pitch Deviation Index	Hip	FG	3.09 [-2.55, 8.73]	0.24 [-0.18, 0.65]
Pitch Deviation Index	Hip	Female	0.25 [-6.16, 6.67]	0.02 [-0.39, 0.43]
Pitch Deviation Index	Hip	Male	-4.85 [-16.95, 7.25]	-0.20 [-0.66, 0.27]

**Indicator**	**Segment**	**Group**	**Mean Difference with 95%CI**	**Cohen's d with 95%CI**
Pitch Deviation Index	Hip	SG	-8.48 [-20.58, 3.62]	-0.35 [-0.82, 0.13]
Pitch Deviation Index	Shoulder	FG	2.42 [-8.67, 13.52]	0.10 [-0.32, 0.50]
Pitch Deviation Index	Shoulder	Female	-1.58 [-12.17, 9.00]	-0.06 [-0.47, 0.34]
Pitch Deviation Index	Shoulder	Male	-8.40 [-21.57, 4.78]	-0.32 [-0.79, 0.16]
Pitch Deviation Index	Shoulder	SG	-13.51 [-24.66, -2.37]	-0.60 [-1.10, -0.09]
Pitch Frequency	Head	FG	-0.25 [-0.37, -0.14]	-0.96 [-1.45, -0.46]
Pitch Frequency	Head	Female	-0.28 [-0.40, -0.16]	-1.00 [-1.49, -0.49]
Pitch Frequency	Head	Male	-0.18 [-0.30, -0.06]	-0.74 [-1.25, -0.21]
Pitch Frequency	Head	SG	-0.21 [-0.35, -0.07]	-0.77 [-1.29, -0.23]
Pitch Frequency	Hip	FG	-0.07 [-0.14, -0.01]	-0.51 [-0.94, -0.07]
Pitch Frequency	Hip	Female	-0.10 [-0.16, -0.03]	-0.66 [-1.10, -0.20]
Pitch Frequency	Hip	Male	-0.12 [-0.21, -0.03]	-0.64 [-1.15, -0.13]
Pitch Frequency	Hip	SG	-0.15 [-0.24, -0.06]	-0.83 [-1.36, -0.28]
Pitch Frequency	Shoulder	FG	-0.07 [-0.13, -0.00]	-0.46 [-0.89, -0.02]
Pitch Frequency	Shoulder	Female	-0.10 [-0.16, -0.03]	-0.66 [-1.10, -0.20]
Pitch Frequency	Shoulder	Male	-0.06 [-0.14, 0.01]	-0.40 [-0.88, 0.09]
Pitch Frequency	Shoulder	SG	-0.10 [-0.18, -0.02]	-0.63 [-1.13, -0.12]
Pitch Time	Head	FG	0.11 [0.05, 0.17]	0.81 [0.34, 1.28]
Pitch Time	Head	Female	0.15 [0.09, 0.22]	1.01 [0.50, 1.51]
Pitch Time	Head	Male	0.11 [0.03, 0.19]	0.71 [0.18, 1.22]
Pitch Time	Head	SG	0.17 [0.08, 0.26]	0.97 [0.40, 1.52]
Pitch Time	Hip	FG	0.03 [-0.01, 0.07]	0.29 [-0.13, 0.70]
Pitch Time	Hip	Female	0.03 [0.00, 0.07]	0.44 [0.00, 0.86]
Pitch Time	Hip	Male	0.05 [-0.01, 0.11]	0.44 [-0.05, 0.92]
Pitch Time	Hip	SG	0.06 [0.01, 0.11]	0.63 [0.11, 1.13]
Pitch Time	Shoulder	FG	0.01 [-0.00, 0.02]	0.38 [-0.05, 0.80]
Pitch Time	Shoulder	Female	0.03 [-0.00, 0.06]	0.39 [-0.04, 0.81]
Pitch Time	Shoulder	Male	0.03 [-0.01, 0.06]	0.40 [-0.08, 0.88]
Pitch Time	Shoulder	SG	0.05 [0.00, 0.10]	0.50 [0.00, 0.98]
Pitch Velocity	Head	FG	48.41 [30.48, 66.33]	1.17 [0.63, 1.69]
Pitch Velocity	Head	Female	53.01 [36.34, 69.68]	1.38 [0.79, 1.94]

Indicator	Segment	Group	Mean Difference with 95%CI	Cohen’s d with 95%CI
Pitch Velocity	Head	Male	49.20 [21.48, 76.92]	0.88 [0.33, 1.42]
Pitch Velocity	Head	SG	55.08 [28.82, 81.34]	1.04 [0.46, 1.61]
Pitch Velocity	Hip	FG	3.04 [0.17, 5.92]	0.46 [0.02, 0.88]
Pitch Velocity	Hip	Female	2.02 [-1.37, 5.41]	0.26 [-0.16, 0.67]
Pitch Velocity	Hip	Male	0.60 [-4.96, 6.17]	0.05 [-0.41, 0.52]
Pitch Velocity	Hip	SG	-0.70 [-6.60, 5.20]	-0.06 [-0.52, 0.40]
Pitch Velocity	Shoulder	FG	9.89 [5.14, 14.64]	0.90 [0.41, 1.38]
Pitch Velocity	Shoulder	Female	10.25 [5.68, 14.82]	0.97 [0.46, 1.46]
Pitch Velocity	Shoulder	Male	11.12 [4.69, 17.55]	0.86 [0.31, 1.39]
Pitch Velocity	Shoulder	SG	11.58 [5.40, 17.76]	0.93 [0.37, 1.48]
